# Water Rewrote the Rules: Why There is no Universal Setting in Underwater Endoscopy

**DOI:** 10.1055/a-2979-6011

**Published:** 2026-10-21

**Authors:** Antonio Capogreco, Cesare Hassan, Alessandro Repici

**Affiliations:** 1 Gastroenterology, Endoscopy Unit 9268 IRCCS Humanitas Research HospitalRozzanoMilanItaly; 2 Department of Biomedical Sciences 437807 Humanitas UniversityPieve Emanuele, MilanItaly; 3 Department of Biomedical Sciences 437807 Humanitas UniversityMilanItaly

## 

Few technical shifts of the last decade have changed endoscopic resection as profoundly, or as quickly, as filling the operative field with water. Optical resolution improves, smoke disappears, buoyancy opens the planes for us, and blood and debris are carried away instead of accumulating. 1 These are not impressions: underwater ESD is, to date, the only strategy shown in a network meta-analysis of randomised trials to deliver superior dissection speed compared with both traction-assisted and conventional ESD, with equivalent R0 rates and a favourable adverse-event profile. 2 In the colorectum, it has brought otherwise forbidding lesions within reach, with less distension and greater haemodynamic stability through long procedures. 3 Intraprocedural bleeding, the event that most often derails a dissection, falls from 29.9% to 6.3% per vessel when vessels are pre-sealed under immersion. 4 A field that stays clean is a field in which a trainee can actually be taught. Immersion has changed endoscopy.

But its most far-reaching consequence is not optical. It is electrical, and it has been almost entirely underestimated. Every current we use was conceived and calibrated for a circuit in which the electrode is surrounded by gas, an insulator that confines the current to the point of contact and makes the thermal effect predictable. Immersion abolishes that insulator and makes the medium itself part of the circuit, so that the whole vocabulary of electrosurgery (effect, peak voltage, spark, arc, power) comes to describe a circuit that no longer exists. The magnitude is not subtle: moving from air to saline reduces impedance by roughly 99%, reduces measured peak voltage by 52–78%, and increases delivered power by 237–2030%. 5 6 A mode that cuts in gas may fail to coagulate in water; a mode that sparks violently may fall silent; and a mode that gives an immediate frozen effect may give nothing at all. Underwater endoscopy did not adjust our currents; it rewrote their universe. The response has been improvisation: settings invented operator by operator, none externally validated, leaving a field of private protocols and almost no shared evidence about what any of them delivers.

Against that background, the report by Do and colleagues is a welcome contribution. 7 They describe a stepwise workflow for oesophageal underwater third-space endoscopy, using Endo Cut I (Effect 4) for mucosal entry and myotomy, Soft Coag (Effect 4, 70 W) as the workhorse mode, and Forced Coag (Effect 3, 60 W) in reserve, applied to 40 consecutive procedures with 100% technical success and no severe adverse events. Forced Coag was used in two early cases and abandoned, because it filled the field with gas.

Two aspects deserve credit. The settings are written down for every phase, and are therefore testable by others. The attention to bubbles is well placed: in an immersed tunnel, they interrupt the saline–electrode interface and can abolish the pre-sealing effect on which safe vessel management depends, which continuous flushing restores by renewing that interface. 8

Why, then, does the interpretation remain limited? First, the mechanistic anchor is a set of manufacturer-specified nominal peak voltages, and nominal specifications describe a generator, not a circuit. No electrical measurement was performed, and the chosen parameter is the one least stable between the two media 6 ; the claimed alignment between declared voltages and intraoperative behaviour is a hypothesis presented as a finding.

Second, the physics tells a more interesting story than the protocol allows. Bubbles are the visible signature of sparking, and sparking requires enough voltage to strike an arc. Soft Coag is voltage-limited below that threshold by design: it will not arc, and will not bubble, in gas or in water. That is a property of the waveform, not an effect of immersion. The corollary is less comfortable, since immersion depresses delivered voltage further and Soft Coag can fall below the level needed for reliable coagulation, which is what Japanese operators compensate for by raising the effect step. Forced Coag is the mirror image: the high peak voltage that lets it arc in CO _2_ collapses under immersion, attenuating the arc. It therefore does not inevitably mean bubbles underwater. Depending on tip dimensions, tissue, contact, and activation pattern, and with the voltage drop exploited by lowering the effect or flushing during activation, it can be brought to coagulate without a bubble screen. 9 10 The window has two edges: within the same POEM, forced coagulation at low effect that gave an immediate frozen effect in CO _2_ gave no tissue effect whatever in saline, while swift coagulation at a higher effect pre-sealed consistently in the same immersed field. 9 The bubbles the authors saw belong to their particular combination of mode, effect, knife, and irrigation regimen, not to Forced Coag underwater as such.

Third, a universal setting is therefore unattainable: the same effect can be reached by different modes in different environments, and a setting optimal in one circuit may be inert in another. 9 The electrode itself is decisive. In a saline bench model, lateral thermal spread increased by 256% with a 1.5-mm knife but by only 71% with 0.4- to 0.5-mm knives, with electrode diameter and immersion emerging as the strongest independent predictors of spread and the impedance and voltage drop far more pronounced at the thicker tip. 11 Contact pressure and dwell time, saline and injectate conductivity, tissue impedance, flow during activation, and the generator’s regulation algorithm all enter the same equation. 12 13 The report illustrates this in passing: Soft Coag is called the principal dissection mode, yet a non-arcing waveform desiccates rather than divides.

Finally, the design constrains what can be concluded. A retrospective, single-operator, single-arm series pooling three heterogeneous procedures demonstrates that a protocol is deliverable, but little more. The abandonment of Forced Coag within the first five cases is inseparable from the learning curve. No endpoint capable of discriminating between energy strategies is reported, and vessel calibre is left undefined, although a threshold such as ≥1.2 mm is feasible. 4

How, then, should the field approach the problem, building on what these authors have started? The logic has to be inverted. We have spent a decade asking which setting to use, as though the answer were a number that could be copied from one endoscopy room to another. The question worth asking is what effect we intend to produce, whether pre-sealing without transection, division without lateral thermal spread or haemostasis without a bubble screen, and which variables must be moved to obtain it in the circuit we are working in. The setting is the output of that reasoning, never its starting point.

Four steps would take us a long way. The first is measurement: bench characterisation of impedance, delivered power, peak voltage, and thermal spread in air versus saline, for the knife–generator combination in use. 6 11 What emerges is not a universal preset but a map of how each mode migrates across effect steps when the medium and the electrode change, so that an operator moving from gas to water knows in which direction, and by how much, to compensate.

The second is reporting the circuit rather than the screen: generator, mode and effect step, power limit, knife model with tip geometry and exposed length, saline and injectate composition, irrigation rate, whether flow runs during activation, and the activation pattern. Do and colleagues have gone further than most here; extending that transparency to the variables that determine the result would let their protocol be reproduced rather than admired.

The third is comparison on endpoints that matter: prospective studies anchored to defined vessel thresholds and bleeding endpoints, reporting coagulation and procedure times, post-procedural coagulation syndrome, and graded adverse events, 14 so that competing strategies can be ranked rather than described in parallel.

The fourth is educational, and perhaps the most consequential. If settings are circuit-dependent, teaching presets teaches the wrong thing. A trainee needs to predict what a waveform will do in a given medium with a given electrode, to recognise from the tissue response that the voltage has collapsed or that an arc has formed, and to reach for the effect step or the flushing pedal accordingly. Existing electrosurgical guidance was written for a gas-filled lumen 13 ; consensus work updating it for immersion is overdue.

Several strategies can reach the same endpoint, and that is a strength rather than a confusion, provided we understand why. Only a thorough knowledge of the currents we use, and of everything in the circuit that modifies them, allows the intended effect to be obtained reliably and reproduced by others. Do and colleagues have published their settings; the task now is to measure what those settings deliver once the tunnel fills with saline.
